# P-2121. A prospective observational study of usefulness of interferon-gamma release assay for latent tuberculosis infection in pediatric hematopoietic stem cell transplant recipients

**DOI:** 10.1093/ofid/ofae631.2277

**Published:** 2025-01-29

**Authors:** Hyejin So, Jina Lee

**Affiliations:** Chungnam National University Sejong Hospital, Sejong, Ch'ungch'ong-namdo, Republic of Korea; Asan medical center, Seoul, Seoul-t'ukpyolsi, Republic of Korea

## Abstract

**Background:**

Pediatric hematopoietic stem cell transplantation (HSCT) recipients are one of the high-risk groups concerning tuberculosis and screening for latent tuberculosis infection (LTBI) prior to HSCT is important and accurate diagnostic tests for predicting LTBI in these high-risk children are on demand. However, there are concerns for Interferon-gamma release assay(IGRA) being less sensitive in the children group for screening LTBI. This study was aimed to evaluate the predictive value of Quantiferon-TB Gold test (QFT-G) using the enzyme-linked immunosorbent spot assay (T-SPOT.TB) in immunocompromised Korean children.

**Methods:**

In this prospective observational study, children under age 20 who were scheduled for allogeneic HSCT at Asan Medical Center Children`s Hospital were enrolled during March 2017 and May 2018. QFT-G test and T-SPOT.TB were done prior to HSCT. Concordance rates were calculated among each group. Electronic Medical Records was reviewed for clinical and demographic data of the enrolled patients.

**Results:**

Among the 62 patients who were planned to receive allogenic HSCT during March 2017 and May 2018, 11 patients were excluded and a total of 51 patients were enrolled. Median age was 10 years and the underlying disease leading to HSCT was mostly aplastic anemia (31.4%). Others were acute myeloid leukemia (23.5%), acute lymphoblastic leukemia (15.7%), myelodysplastic syndrome (7.8%), and other benign disease (17.6%). About 52% of the patients were applied immunosuppressive drugs in the recent 3-month period prior to testing. 4 patients were considered LTBI based on screening test results. At 2 year follow up period, 1 patient was confirmed pulmonary TB. Overall concordance rate was 82.4% (42/51). The age-based concordance rate was 93.8% (14/15) for children younger than 5-years old and 77.8% (28/36) for 5 or above. Concordance rate between negative test results and not TB diagnosis was further checked; QFT-GIT 97.8% (44/45), T-SPOT. TB 97.9% (46/47), and TST 100% (10/10).

**Conclusion:**

This study shows that the GFT-GIT well matched with the T-SPOT.TB for screening LTBI and children under age 5 showed to have higher concordance rates compared to the older age group. This could provide evidence to testing younger children with IGRA assays.

**Disclosures:**

All Authors: No reported disclosures

